# High-Dose Vitamin C Tends to Kill Colorectal Cancer with High MALAT1 Expression

**DOI:** 10.1155/2020/2621308

**Published:** 2020-11-23

**Authors:** Jifei Chen, Fengxian Qin, Yu Li, Shanying Mo, Kaifeng Deng, Yujie Huang, Weijun Liang

**Affiliations:** Medical Science Laboratory, The Fourth Affiliated Hospital of Guangxi Medical University, Liuzhou, Guangxi 545005, China

## Abstract

**Background:**

Vitamin C (Vc) deficiency is frequently observed in cancer sites and has been proposed to have an antitumor effect. However, the mechanism of Vc's killing effect is not clear. Besides, epigenetic alterations exhibit significant effects on colorectal cancer (CRC). This study aimed to explore the mechanism of Vc's killing effect and its association to epigenetic alterations in CRC.

**Methods:**

Cell morphology, apoptosis, proliferation, and cycle were assayed to test Vc's suppressive function in CRC cell lines. Xenograft and peritoneal implantation metastasis models were performed to evaluate the high-dose Vc's inhibitory effect on tumor growth and metastasis. Immunohistochemistry was used to measure CD31 expression in solid tumors. A literature summary was applied for screening differently expressed long noncoding RNAs (lncRNAs) in CRC tissues and was closely associated with CRC progression. The qPCR was used to detect the expression of these lncRNAs. The association between Vc and metastasis-associated lung adenocarcinoma transcript 1 (MALAT1) was evaluated in MALAT1-transfected CRC cells and a xenograft model.

**Results:**

Vc was confirmed to function in proliferation suppression, apoptosis induction, and S phase arresting in CRC cell lines. High-dose Vc, but not physiologically low-dose Vc, was identified as a suppressive function on tumor growth in xenograft models and an inhibitory effect on implantation metastasis in peritoneal implantation metastasis mice. Furthermore, a consistent downregulation of MALAT1 induced by Vc was verified among CRC cell lines and tumor tissues from both mouse models. Finally, experiments on MALAT1-knockdown CRC cells and its xenograft model suggested that Vc had a tendency in killing CRC with high MALAT1 expression.

**Conclusions:**

Our findings demonstrate that high-dose Vc has more efficiency in suppressing CRC with higher MALAT1 expression. It gives high-dose Vc the possibility of a better curative effect on CRC with overexpressed MALAT1. Further clinical studies are still needed.

## 1. Background

Vitamin C (Vc) is an essential micronutrient for humanity, and it is well known for its antioxidant activity [1]. In the early 1970s, high-dose Vc was developed to be a cancer therapy. The cancer patients had an increased survival rate after given high-dose intravenous Vc (IVC, 10 g/day for 10 days) followed by oral delivery [2]. In contrast, Creagan and Moertel et al. demonstrated that the administration of high-dose of Vc had no efficacy in cancer therapy, leaving this issue of therapeutic effectiveness to controversy [3, 4]. After decades, clinical studies have not still demonstrated the benefits of oral Vc. As the mechanisms of IVC usage are distinct from those of oral administration, the plasma Vc concentration could be 200 times higher followed by high-dose IVC administration [5], and the serum concentration of Vc is safe and well tolerated as high as 49 mM [6]. High-dose oral and high-dose IVC administration have to be considered as different therapeutic approaches.

Is there a role for high-dose IVC as an anticancer agent? Mechanism researches have shown that high-dose Vc leads to the formation of ascorbate radicals and peroxides that are selectively cytotoxic to cancer cells [7–9]. Recently, Yun et al. [10] reported that the selective death of cancer cells with KRAS or BRAF mutation is caused by dehydroascorbate (DHA) by a targeting inactivation of glyceraldehyde 3-phosphate dehydrogenase (GAPDH) via the glucose transporter 1. Clinical trials and laboratory-based studies indicated an effect of IVC on the inhibition of tumor growth in mouse xenograft models [11], mitigation of chemotherapy side effects [12], improved survival [13], and quality of life [14]. However, the effects of high-dose IVC on colorectal cancer were rarely reported.

Colorectal cancer (CRC) is the second most common cause of cancer-related death worldwide [15]. A series of studies have demonstrated the role of long noncoding RNA (lncRNA) in the development and metastasis of CRC. These CRC-related lncRNAs have been shown to regulate genes by epigenetic modifications and lncRNA-miRNA and lncRNA-protein interactions and act as miRNA precursors or pseudogenes, which subsequently influence cell proliferation, apoptosis, differentiation, invasion, and metastasis [16, 17]. However, it remains unknown if the expression profile of CRC-associated lncRNAs alters after exposure to high-dose Vc during CRC progression and metastasis.

In this article, we evaluated Vc's suppressive effects on CRC cell lines, mouse xenograft, and implantation metastasis CRC models. Then, we focused on MALAT1 because of its consistent reduction after the treatment of high-dose Vc. In addition, the association between Vc and MALAT1 was evaluated in MALAT1-interfered CRC cells and its xenograft model. Results suggest that MALAT1 has a close association with Vc's suppressive function on CRC, which indicates that high-dose IVC has a possible better outcome in treating CRC with higher MALAT1 expression.

## 2. Methods

### 2.1. Cell Culture and Establishment of Stable MALAT1-Knockdown and Negative Control LS174T Cells

The human CRC cell lines LS174T, HT-29, and SW480 were obtained from Shanghai Institutes for Biological Sciences. The cells were cultured in DMEM (Gibco) or RPMI 1640 medium (Gibco) with 10% fetal bovine serum (Gibco), 100 U/ml penicillin G, and 100 *µ*g/ml streptomycin and incubated at 37°C in a humidified atmosphere containing 5% CO_2_ and 95% air. MALAT1-knockdown (KD) and negative control (WT) CRC cells were constructed by lentiviral transfection according to the method of Liu 2016 [18]. After two weeks from the infection, cells were harvested and qRT-PCR was used to confirm the expression level of MALAT1 in KD and WT cells.

### 2.2. Cell Proliferation, Apoptosis, and Cycle Analysis

To examine the effect of Vitamin C (Vc) on cell proliferation, cells (2 × 10^3^/well) were incubated in a 96-well plate for 24 h, stimulated with different doses of Vc for another 24, 48, or 72 h. Cells were washed three times with PBS, after 10 *μ*l CCK8 (DOJINDO, CCK-8) and 90 *μ*l medium was added, optical density was measured under 450 nm and 630 nm, and cell viability was measured as (OD of Vc treated-OD of blank)/(OD of 0 mM Vc treated-OD of blank). For the cell cycle analysis, cells (1 × 10^6^/well) were incubated in a six-well plate for 24 h, stimulated with different doses of Vc for another 24 h. Cells were washed with PBS three times and then fixed with 75% cold ethanol. After hydrating and washing with PBS, cell cycle analysis was carried out by flow cytometry (FCM, Beckman, Navios) after propidium iodide staining (Multisciences, Cell Cycle Staining Kit). For the apoptosis assay, cells (1 × 10^5^/well) were incubated in a six-well plate for 24 h and stimulated with different doses of Vc for another 24 h. Apoptosis was assayed by FCM after stained with Annexin V and PI (Multisciences, Cell apoptosis kit). Three independent experiments were performed in independent cultures at three different time points.

### 2.3. Xenograft and Peritoneum Implantation Metastasis Model

Athymic nude mice (Balb/c, nu/nu) were from the Laboratory Animal Centre, Guangxi Medical University, China. All the animals were maintained under pathogen-free conditions. The Ethics Committee at The Fourth Affiliated Hospital of Guangxi Medical University approved all animal experiments in our study. Six-week-old nude mice were injected subcutaneously (SC) in the groin with LS174T cells (2 × 10^6^/mouse) in 100 *μ*l saline to establish the xenograft model as described in our previous study [19]. In addition, intraperitoneal injection (IP) of LS174T (2 × 10^6^/mouse) was used to construct the implantation metastasis model. Bodyweight and tumor size were recorded every two days. Vitamin C (high-dose 4 g/kg, low-dose 100 mg/kg) was injected intraperitoneally every two days from one week after tumor implantation to the sacrifice day. The high-dose 4 g/kg was proved to achieve a peak concentration as high as 30 mM in mouse peripheral blood after 90 min–180 min after intraperitoneal injection [11]. Five mice were enrolled in each group. Tumor size was calculated as spherical volume (4/3)∗*π*∗ ((length/2 + width/2 + depth)/3) 3.

### 2.4. Humane Endpoints

The health and behavior of experimental mice were monitored every day. The duration of the xenograft and peritoneum implantation metastasis model was 30 days and 26 days, respectively. When the tumor exceeds the midline of the mouse abdomen or the mouse is extremely emaciated or has dyskinesia or exploration disorder, it is defined as the humane endpoint. When the humane endpoint or the experimental endpoint was reached, the mice were sacrificed by intraperitoneal injection of pentobarbital anesthesia.

### 2.5. qPCR, Relative Quantification

The total RNA of Cells and tumor tissues were extracted by RNAiso Plus reagent (TaKaRa). PrimeScript™ RT Reagent Kit with gDNA Eraser (TaKaRa) was used for reverse transcription. SYBR® Premix Ex Taq™ II (TaKaRa) was used in the real-time PCR reaction (7500 Real-Time PCR Instrument, Life). The entire process was carried out according to the manufacturer's instructions. Primers used to amplify the genes were listed in Table S1. The 20 *μ*l PCR reaction contained SYBR green, ROX, primers, cDNA, and RNase free water. PCR reaction condition was as follows: holding at 95°C for 10 minutes, 40 cycles at 95°C for 15 seconds, and 60°C for 60 seconds. PPIA, another frequently used internal reference in qPCR, was used to avoid inaccuracy as Vc could influence the expression of GAPDH [10, 20]. The relative quantification was calculated by −2^ΔΔCT^ or −2^ΔCT^. Each analysis was repeated three times.

### 2.6. Immunohistochemistry (IHC)

IHC was performed according to the instruction of the Abcam IHC procedure. Generally, 4 *μ*m thick paraffin sections were used in this study. After the section, the slides were dewaxed in xylene and rehydrated in graded ethanol. Endogenous peroxidase activity was blocked by 0.3% H_2_O_2_ for 10 minutes. Antigen retrieval was carried out by heat mediation citrate buffer (0.01 M). Rabbit anti-mouse CD31 (Abcam) was used as the primary antibody, and the goat anti-rabbit antibody (ZSGB-BIO) was used as secondary, followed by diaminobenzidine (DAB) visualization. The CD31 positive area and density were measured by Image-Pro Plus 6.0 in two random photos for every slice.

### 2.7. Statistical Analyses

Statistical testing was performed by Student's *t*-test, one-way ANOVA, or two-way ANOVA test with multiple comparisons by Graph pad Prism 7 unless otherwise indicated. Statistical significance was assumed at *p* < 0.05.

## 3. Results

### 3.1. Vitamin C Induces Apoptosis, Proliferation Suppression, and Cycle Arrest in CRC Cells

**Figure 1:**
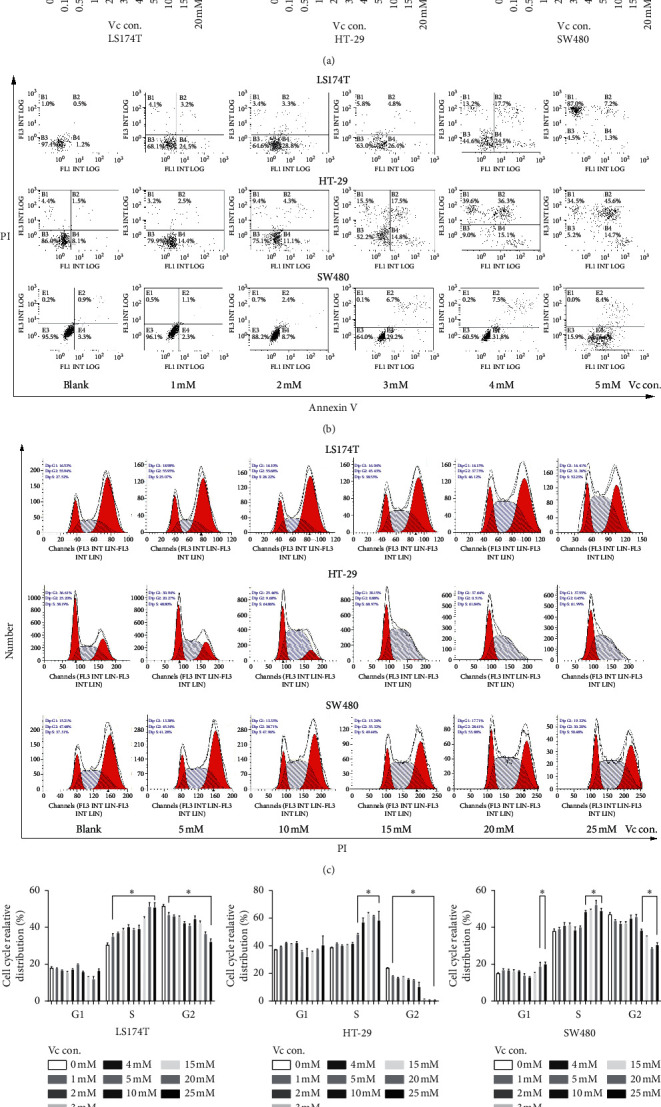
Vitamin C induces cell apoptosis, proliferation suppression, and cycle arrest in CRC. (a) Proliferation test by CCK8 assay. The proliferation of LS174T, HT-29, and SW480 was suppressed by Vc in a dose-dependent manner. (b) The apoptosis test performed by flow cytometry. The apoptosis of LS174T and HT-29 and SW480 was induced by Vc in a dose-dependent manner. The portion of late apoptosis was significantly increased when the concentration of Vc was greater than 3 mM. It should be noted that cell lysis of LS174T and SW480 was observed when cells were treated with 5 mM Vc. (c) Cell cycle analyzed by flow cytometry. The proportion of S phase was significantly increased in LS174T, HT-29, and SW480 cells after the cells were treated with different doses of Vc. (d) Column graphs of the cell cycle calculated after three independent cell cycle experiments. In (a) and (d), ^*∗*^*p* < 0.05 and ^*∗∗*^*p* < 0.01 when the mean of each column was compared to the mean in the group treated with 0 mM Vc. Vc con.: concentration of Vitamin C. Each experiment was performed three times.

The killing effect of Vc was tested in our own CRC cell lines LS174T, HT-29, and SW480. After incubated with different doses of Vc for 4 h, most of the LS174T cells were floated (Figure S1A), and almost all SW480 cells were puffed up to a sphere (Figure S1B). Then, a series of in vitro experiments were performed. The proliferation of these three CRC cell lines was inhibited by Vc in a dose-dependent manner at a concentration of 2 to 3 millimoles (Figure 1(a)). Besides, the apoptosis of LS174 T, HT-29, and SW480 was induced by Vc in a dose-dependent manner (Figure 1(b)). It should be noted that when LS174 T was treated with Vc under a concentration of 5 mM, most of the cells were floated and disrupted, leading to 87% in area B1 (Figure 1(b), top right). Finally, after evaluating the cell cycle, we found that Vc had a significant S phase arresting function in LS174T, HT-29, and SW480 in a dose-dependent manner (Figures 1(c) and 1(d)).

### 3.2. High-Dose Vitamin C Suppresses CRC Growth, Angiogenesis, and Metastasis in Mouse Xenograft and Intraperitoneal Implantation Metastasis Model

**Figure 2:**
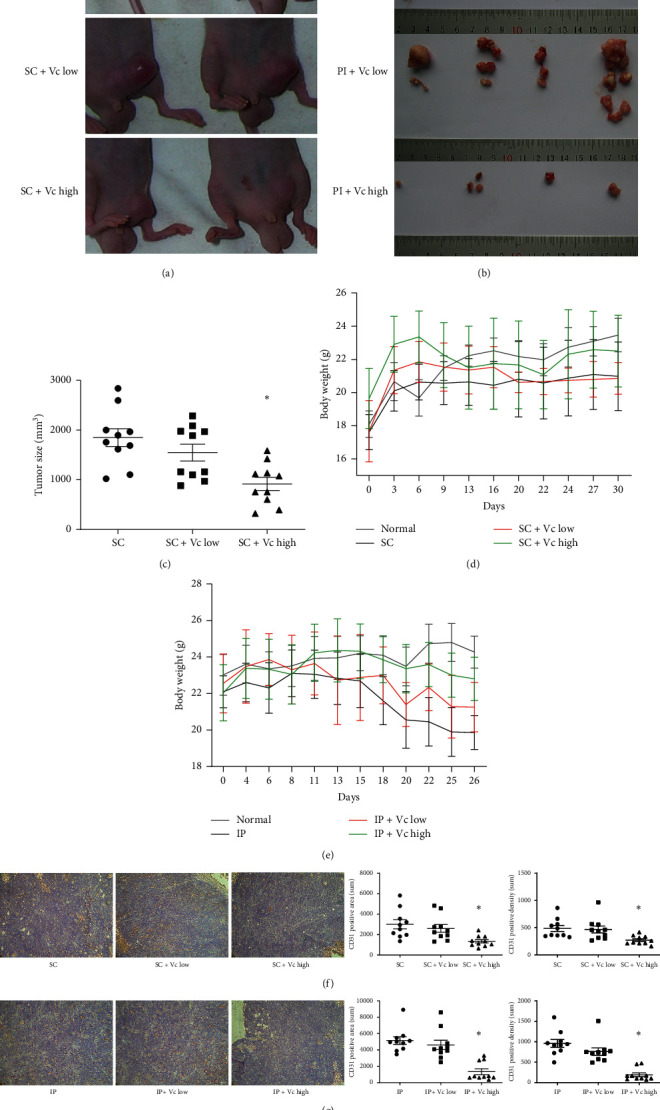
High-dose Vitamin C suppresses CRC growth, angiogenesis, and metastasis in mouse xenograft and intraperitoneal implantation metastasis models. (a) General observation of mice in different xenograft groups (SC, SC + Vc low, SC + Vc high) on the sacrificed day. And the tumor size was calculated in a scatter plot (c). (b) The tumor number and size of different groups in the model of intraperitoneal implantation metastasis (IP, IP + Vc low, IP + Vc high). The body weight change in the tumor growth process in xenograft groups (d) and the tumor metastasis process in the intraperitoneal implantation metastasis groups (e). Only a tendency could be obtained due to the individual differences, weighing equipment, and the inevitable mouse movement during the weighing process. The expression of CD31 in solid tumors in xenograft (f) and implantation metastasis model (g) was detected by IHC, and the right scatter plot indicated the CD31 positive area and density in each slice calculated by Image-Pro Plus 6.0. In (c), ^*∗*^*p* < 0.05 when the tumor size in the SC + Vc high group was compared to that in the SC or SC + Vc low group. In the right scatter plot of (f) and (g), ^*∗*^ means *p* < 0.05 when the SC + Vc high group was compared to the SC or SC + Vc low group, and the IP + Vc high group was compared to the IP or IP + Vc low group . Xenograft groups: SC (Saline), SC + Vc low, and SC + Vc high; intraperitoneal implantation metastasis groups: IP (Saline), IP + Vc low, and IP + Vc high.

After the suppressive effect of high-dose Vc on CRC cell lines was confirmed, we further tested its function in vivo. In our previous work, we successfully established a mouse xenograft (SC) and intraperitoneal implantation metastasis (IP) model [19]. In the SC model, mice treated with high-dose Vc (SC + Vc high) had smaller tumor size (Figures 2(a) and 2(c)) with less body weight loss (Figure 2(d)) during the modeling process. Meanwhile, rare metastasis nodes were found in enterocoelia in the IP + Vc high group (Figure 2(b)). In addition, mice were with bodyweight loss in the IP model (Figure 2(e)). The low-dose Vc used in this study was defined as the physiology dosage according to the instruction of weight conversion for mice. Little differences in tumor burden or bodyweight loss were found between low-dose Vc groups (SC + Vc low, IP + Vc low) and positive control groups (SC, IP), respectively, which indicated that the suppressive function of Vc in tumor growth could only be observed in high dose.

When we were collecting tumor samples from the sacrificed mice, the general observation of the SC model was shown in Figure 2(a). In the IP model, significant bloody ascites were observed in 4/5 IP and 3/5 IP + Vc low group during the surgery process. And in the IP and IP + Vc low groups, most of the metastasis nodes were distributed along the celiac trunk behind the stomach and were hard to dissociate. Meanwhile, no ascitic fluid was observed in the IP + Vc high group, and most metastasis nodes in this group were in the mesentery and were easy to dissociate. These descriptions declared a full record of the surgery process, which suggests that high-dose Vc has an invasion suppressing ability in CRC implantation metastasis (data not shown). Besides, remarkable vessels could be observed under the surface of the xenograft tumors and around the metastasis nodes in the low-dose Vc groups (SC + Vc low, IP + Vc low) and positive control groups (SC, IP) in both mouse models (data not shown). Then, IHC was performed to objectively measure the CD31 expression in both models. Consistent with the general observation, a lower CD31 expression (positive area and density) was verified in both high-dose Vc groups (SC + Vc high, IP + Vc high), which indicated less angiogenesis (Figures 2(f) and 2(g)).

### 3.3. Vitamin C Induces a Stable Downregulation of MALAT1 in CRC In Vitro and In Vivo

**Figure 3:**
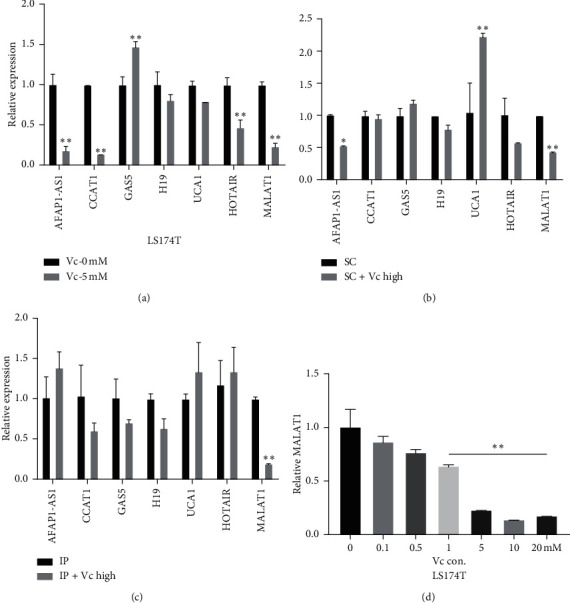
Vitamin C induces a stable downregulation of MALAT1 in CRC in vitro and in vivo. (a) The relative expression of seven lncRNAs was detected by qPCR after LS174T was treated with 5 mM Vc. The relative expression of the lncRNA profile was detected in solid tumor tissues acquired from models of xenograft (b) and implantation metastasis (c). (d) The relative expression of MALAT1 was detected by qPCR after LS174 T was treated with different doses of Vc as final verification. In (a), ^*∗∗*^*p* < 0.01 when the expression of different lncRNAs in 5 mM Vc was compared to that in 0 mM group. In (b), ^*∗*^*p* < 0.05 and ^*∗∗*^*p* < 0.01 when the expression of different lncRNAs in the SC + Vc high group was compared to that in the SC group. In (c), ^*∗∗*^*p* < 0.01 when the expression of different lncRNAs in the IP + Vc high group was compared to that in the IP group. In (d), ^*∗∗*^*p* < 0.01 when the expression of MALAT1 in 1, 5, 10, and 20 mM groups was compared to that in 0 mM group.

We next asked whether the lncRNAs that are associated with CRC development, metastasis, and prognosis [17] account for the suppressive effect of Vc. Firstly, we detected the expression of seven lncRNAs (Table S2), which were closely related to CRC progression and were verified in LS174T in recent reports. Surprisingly, AFAP1-AS1, CCAT1, HOTAIR, and MALAT1, highly expressed in CRC, showed a remarkable reduction when treated with 5 mM Vc. By the way, H19 and UCA1 only showed a reduction trend. GAS5, which proved a downregulation in CRC, was increased after Vc treatment (Figure 3(a)).

As the working environment of Vc differed greatly in vivo compared to that in vitro, we shifted our focus to detecting these lncRNAs in the tumor burden mice, the peritoneum implantation metastasis model (IP), and xenograft model (SC). Not surprisingly, the changing pattern of these lncRNAs showed a great difference under the impact of the tumor environment in vivo. In the tumor tissues of SC groups (Figure 3(b)), MALAT1 and AFAP1-AS1 showed a significant reduction after being treated with high-dose Vc, while the expression of UCA1, which showed a reduction trend in vitro, was increased in SC tumor tissue after high-dose Vc. In the tumor tissues of IP groups (Figure 3(c)), only the expression of MALAT1 showed a significant reduction in the high-dose Vc group. It should be noted that the low-dose Vc, defined as a physiological dose, showed no effect on the lncRNA alteration in tumors from both mouse models (data not shown).

Then, we compared the different change patterns of these seven lncRNAs among cells and solid tumor samples from the SC or IP model. Only MALAT1 showed a stable and consistent reduction in CRC when treated with high-dose Vc. Finally, we detected the expression of MALAT1 after LS174T was treated with different doses of Vc for final confirmation. And MALAT1 was significantly downregulated in a dose-dependent manner after being treated with Vc at a concentration as low as 1 mM (Figure 3(d)).

### 3.4. Vitamin C Tends to Kill Colorectal Cancer with Higher MALAT1 Expression In Vitro

**Figure 4:**
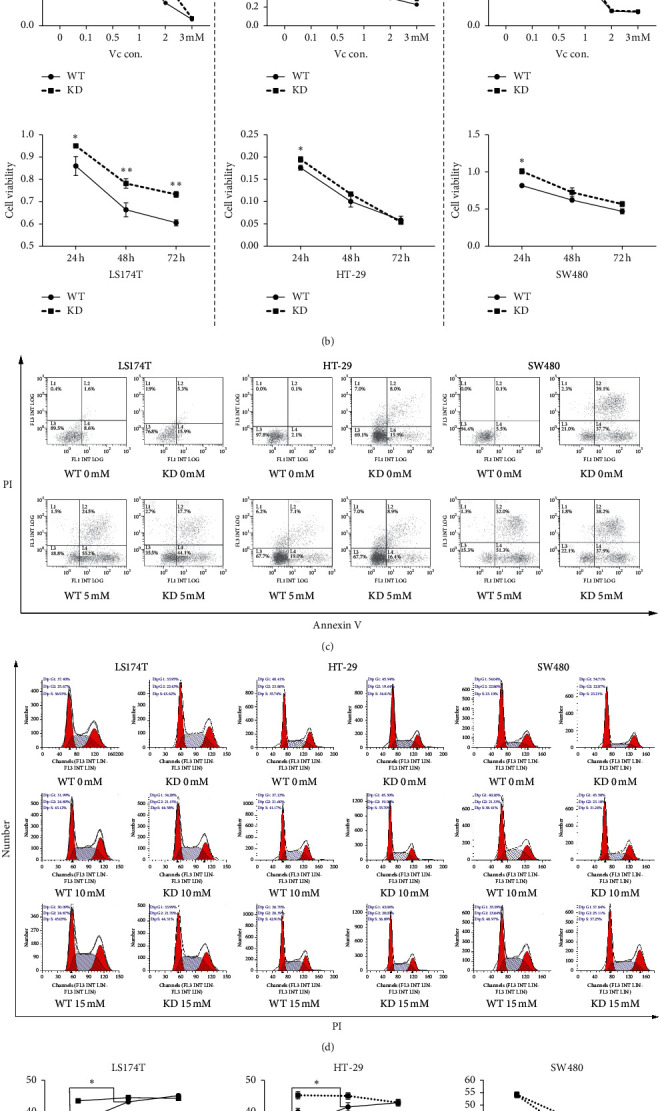
Vitamin C selectively kills colorectal cancer with higher MALAT1 expression in vitro. (a) The relative MALAT1 expression in LS174T, HT-29, and SW480 after lentiviral transfection. Negative control (WT), MALAT1-knockdown (KD). (b) The proliferation of LS174T, HT-29, and SW480 was measured in WT and KD groups treated with 0, 0.1, 0.5, 1, 2, and 3 mM Vc for 24 h (up) and 1 mM Vc for 24, 48, and 72 h (down). (c) The apoptosis test was performed in WT and KD cells under 0 mM and 5 mM Vc. (d) The cell cycle of LS174T, HT-29, and SW480 cell lines was evaluated in the WT and KD groups treated with 0, 10, and 15 mM Vc. The portion of G1, S, and G2 was labeled on the left top of each cycle graph. (e) The portion of G1, S, and G2 was calculated after three independent tests and shown in line chart. In (a), ^*∗∗∗∗*^*p* < 0.0001 when the expression of MALAT1 in the KD group was compared to that in the WT group. In (b), ^*∗*^*p* < 0.05 and ^*∗∗*^*p* < 0.01, when the cell viability was compared between WT and KD groups at 1 and 2 mM Vc in LS174 T, at 1 mM in HT-29, and at 0.5 mM and 1 mM in SW480 ((b) up) or at 24, 48, and 72 h in LS174T, at 24 h in HT-29, and at 24 h in SW480 ((b) down). In (e) ^*∗∗*^*P* < 0.01 and ^*∗*^*p* < 0.05, when the portion of S phase was compared among the 0 mM, 10 mM, and 15 mM groups.

To figure out the role of MALAT1 in the suppressive process of high-dose Vc. The proliferation, apoptosis, and cycle were reevaluated in both negative control (WT) and MALAT1-knockdown (KD) groups. Lentivirus transfection reduced the expression of MALAT1 in LS174T, HT-29, and SW480 cells by 75%, 62%, and 50%, respectively (Figure 4(a)).

In the proliferation experiment, the sensitivities to Vc's killing effect in both WT and KD cells were analyzed after cells were treated with different doses of Vc for 24 h (Figure 4(b), up). A more suppressive rate was observed in WT cells compared to KD cells, and the difference was significant under 1 mM or 2 mM Vc in LS174T, 1 mM Vc in HT-29, and 0.5 mM or 1 mM Vc in SW480. Next, cells were treated with 1 mM Vc for 24, 48, and 72 h for further proliferation test. The WT cells, which had higher MALAT1 expression, were more sensitive to Vc's killing effect compared to KD cells at different time points in different kinds of cells (LS174T at 24, 48, 72 h, HT-29 at 24 h, and SW480 at 24 h) (Figure 4(b), down).

When we tested the possible different apoptosis states under Vc treatment in WT and KD cells, the apoptosis portion in KD cells was a litter higher than that in WT cells without Vc treatment. Surprisingly, 5 mM Vc significantly induced more apoptosis in the WT cells (WT 5 mM compared to WT 0 mM) than in the KD group (KD 5 mM compared to KD 0 mM) in LS174T, HT-29, and SW480 cell lines, while the early or late apoptosis portion showed little difference between KD 0 mM and KD 5 mM in HT-29 and SW480 cell lines (Figure 4(c)). As shown previously, Vc could induce S phase arrest in CRC cells (Figures 1(c) and 1(d)). When this effect was assessed in KD cells, Vc was found to be more efficient in arresting WT CRC cell lines in S phase (Figure 4(d) and 4(e)), while there was rarely any change in G1, S, or G2 portions in KD group in LS174 T and HT-29 cells after KD cells were treated with different doses of Vc. In SW480 cells, there was a significant difference in the S phase and G1 phase when WT 10 mM was compared to WT 0 mM and KD 10 mM was compared to KD 0 mM, respectively. What needed to be pointed out was that 10 mM Vc induced more S and G1 phases changed in the WT SW480 group. These results indicated that Vc was more efficient in killing CRC cells with relatively higher MALAT1 expression.

### 3.5. Vitamin C Tends to Kill Colorectal Cancer with Higher MALAT1 Expression In Vivo

**Figure 5:**
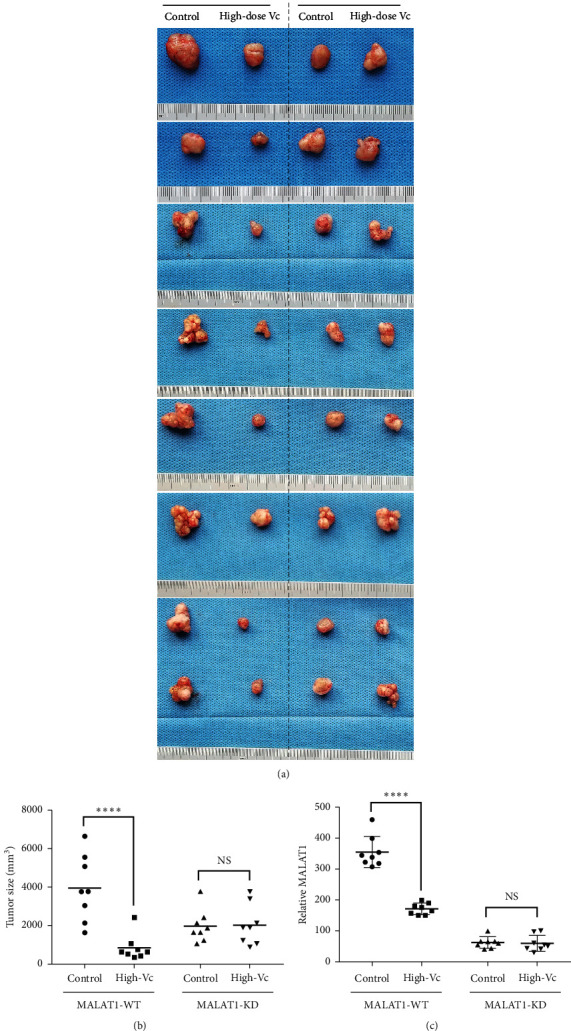
Vitamin C tends to kill colorectal cancer with higher MALAT1 expression in vivo. (a) Different sizes of tumors in MALAT1-WT and MALAT1-KD mice after treatment with saline (control) or high-dose Vc. (b) The tumor sizes were calculated after the mice in different passage groups were treated with saline or high-dose Vc for 28 days. (c) The MALAT1 expression of solid tumor tissues. In (b) and (c), ^*∗∗∗∗*^*p* < 0.0001 when the tumor size or MALAT1 expression in the high-Vc group was compared to that in the control group in the MALAT1-WT mice. NS stands for no significant difference.

After Vc was verified to be more efficient in killing CRC cells with high MALAT1 expression in vitro, we then focused on the in vivo experiment. MALAT1-knockdown (KD) and negative control (WT) LS174T cells (2 × 10^6^/mouse, 100 *μ*l saline, 16 mice per group) were injected subcutaneously (SC) in the groin. At one week after the injection, mice (KD and WT) were then intraperitoneally injected with saline (control group) or 4 g/kg Vc (high-Vc group) once every two days. In the WT groups, tumors in the high-dose Vc group were significantly smaller than those in the control group (Figures 5(a) and 5(b)), while in the KD groups, tumor size had no obvious difference between high-dose Vc and control groups (Figures 5(a) and 5(b)). Furthermore, the MALAT1 expressions in the tumors from WT mice were significantly downregulated in the high-dose Vc group, while no significant difference was found between control and high-dose Vc groups in KD mice (Figure 5(c)).

## 4. Discussion

In CRC patients, the mutation state of KRAS and BRAF is consistent in situ with CRC and corresponding metastatic foci, and the effect of chemotherapeutic drugs such as antiepidermal growth factor receptor antibodies, cetuximab, and panitumumab is also affected by the mutation state of KRAS and BRAF [21]. Yun's article stating that Vc selectively kills KRAS and BRAF mutant CRC [10] makes us regain confidence in the study of Vc, a controversial cancer suppressor [22]. As the effect of IVC was rarely reported in CRC, we verified the function of Vc in CRC cells at different doses. In the beginning, interestingly notable morphological alteration and flotage were found in SW480 and LS174T cell lines (Figures S1A and S1B) only after 4h of incubation with high-dose Vc, while no noteworthy change was observed in HT-29 cells in general (data not shown). To confirm the effects of Vc on CRC cells, we further performed cell proliferation (Figure 1(a)), apoptosis (Figure 1(b)), and cycle analysis (Figures 1(c) and 1(d)). Then, Vc was found to have a significant dose-dependent suppressive effect on proliferation and apoptosis-inducing ability at early or late stages, and S phase arrest function in CRC cell lines, which accorded to previous experiments performed in HT-29, BLM, SK-MEL-28, and HUH7 cells from other cancer types [23–25]. These in vitro results led us to further explore the ability of Vc in vivo.

Previous works had proved that intraperitoneal injection of high-dose Vc (4 g/kg) could increase Vc in mouse blood to a peak concentration of more than 30 mM after 90–180 min and did not produce any obvious adverse reaction [11, 26], thus avoiding repeated tail vein injections. We then set groups of high dose (4 g/kg), low dose (100 mg/kg, physiological dose), and positive control (saline) to evaluate the suppressive function of Vc in nude mouse xenograft (SC) and intraperitoneal implantation metastasis (IP) models. In the xenograft model, mice in the high-dose Vc group (SC + Vc high) had smaller tumor size and less bodyweight loss. This suppressive effect on the tumor was not the first discovery but only a complement to previous works. To be specific, it declared a suppressive effect of high-dose Vc on a nude mouse xenograft model established by cell lines of ovarian cancer, pancreatic cancer, glioblastoma [11], and mesothelioma [27]. Meanwhile, we established another model by injecting CRC cells directly into the peritoneal to mimic the implantation metastasis. After about one-month growth, rare metastasis nodes were explored in enterocoelia during surgery in the high-dose Vc group (IP + Vc high), while more metastasis nodes with bigger sizes were significantly found in the positive (IP) and low-dose groups (IP + Vc low). Besides, bloody ascites or liver surface implantation (data not shown) was found in some mice in IP and IP + Vc low groups, which did not exist in the IP + Vc high group. And it needed to be pointed out that no significant difference was observed between the positive control (SC and IP) and low-dose Vc (SC + Vc low and IP + Vc low) groups, respectively (data not shown). Another key factor for tumor progression and metastasis is angiogenesis. The CD31, also known as platelet endothelial cell adhesion molecule, which was normally expressed on endothelial cells, was evaluated in solid tumor tissues collected from mouse models by IHC-P. In accordance with the general view of vessel distribution during section, a significant decrease of CD31 expression was found in high-dose Vc groups in SC and IP models. In summary, high-dose Vc was confirmed to be an inhibitor in the xenograft model, and this was the first time that high-dose Vc, not the physiological low-dose Vc, declared a suppressive effect on implantation metastasis, which provided a new strategy in reducing the implantation metastasis rate during the CRC invasion progress or surgical disseminated planting. However, it still needs to be proved by further preclinical studies and clinical trials.

After years of study, accumulating evidence suggests that lncRNAs play important roles in tumor progression. In our study, we established a small lncRNA profile after the literature summary, which contained seven lncRNAs involved in CRC development and progression, sustaining proliferation, metastasis, and invasion. To understand the relationship between lncRNA and Vc treatment, the expression of this lncRNA profile, which was closely related to CRC cell proliferation and cell cycle [17, 28] was detected in LS174T under the treatment of 5 mM Vc (Figure 3(a)). Intriguingly, the expressions of lncRNAs that are highly expressed in CRC as previously reported were significantly decreased after Vc treatment, while GAS5, previously reported to be downregulated in CRC, was found to be upregulated. Thus, the alteration of these functional lncRNAs under Vc treatment might be a possible explanation for the effects in proliferation suppression, apoptosis induction, cell cycle arresting effects in vitro, and the ability in solid tumor growth and metastasis inhibition in vivo.

To further evaluate the change of lncRNA profile in vivo, the expression of these seven lncRNAs was also measured in solid tumor samples from mouse xenograft and implantation metastasis models by qPCR. The results were not as expected and were shown in Figures 3(b) and 3(c). The expressions of upregulated lncRNAs involved in CRC development and progression (CCAT1, MALAT1, and UCA1), sustaining proliferation (CCAT1, MALAT1, and UCA1), metastasis and invasion (CCAT1, H19, and HOTAIR), and clinical application (AFAP1-AS1, H19) were rarely altered in the solid tumor from SC and IP mice with high-dose Vc treatment. The alteration of GAS5, which was downregulated in the separated CRC process, declared no statistical significance. Fortunately, the downregulation of MALAT1 was verified and was consistent among CRC cell samples and solid tumor samples from SC and IP models after high-dose Vc treatment. Then, the expression of MALAT1 was redetected after LS174T was treated with different doses of Vc. And MALAT1 was found to be downregulated significantly in a dose-dependent manner of Vc. This suggested that high-dose Vc could have a possible epigenetic therapeutic function in CRC. Therefore, further experiments were designed for exploring the association between Vc and MALAT1.

MALAT1 was one of the first human lncRNAs identified in metastatic lung cancer cells [29]. Its elevated expression was associated with metastasis and reduced overall survival in patients with multiple tumor types [30]. Subcutaneous injection of MALAT1 antisense oligonucleotides could effectively downregulate the MALAT1 expression in the tumor site and result in a formation of cystic and poorly metastasizing tumors in the luminal B breast cancer mouse model [31]. As the expression of MALAT1 could be reduced by high-dose Vc treatment, we in turn explored the role MALAT1 played in the killing function of Vc. Surprisingly, Vc was found to be more efficient in proliferation suppressing, apoptosis inducing, and S phase arresting in WT (lentiviral transfection, negative control) CRC cells, while no obvious effect was found in KD (lentiviral transfection, MALAT1-knockdown) CRC cells in vitro (Figure 4), which indicated that MALAT1 is an important intermediate link in the Vc's suppressive function in CRC. Moreover, this suppressive function of Vc was analyzed in a xenograft constructed by lentiviral transfected LS174T cells. The suppressive effect of high-dose Vc on the MALAT1-WT xenografts was found to be more efficient (Figure 5). Meanwhile, no significant suppressive function was observed in MALAT1-KD xenografts. These in vitro and in vivo works demonstrated that high-dose Vc could be a new promising therapeutic agent for the treatment of CRC patients whose tumor was at the high MALAT1 expression level.

## 5. Conclusions

In summary, the effects of Vc in proliferation suppression, apoptosis induction, and S phase arrest are verified in CRC cell lines, and the function of high-dose Vc in tumor progression suppression is confirmed by our mouse xenograft model, where these in vitro and in vivo results are in accordance with previous reports tested in multiple kinds of tumor cells, including few CRC cell lines. In addition, this is the first time that high-dose Vc, but not physiological low-dose Vc, is proved to have the inhibitory function in implantation metastasis in the mouse model, which provides a new strategy in reducing the implantation metastasis rate during the CRC invasion progress or surgical disseminated planting. Meanwhile, this is the first time that Vc, the contradictory agent, declares an association with MALAT1 downregulation, an epigenetic alteration in tumor cells. And surprisingly, high-dose Vc is found to be more efficient in killing CRC cells and tumors from xenografts with relatively high MALAT1 expression, which indicates that MALAT1 could be a new marker in directions of using high-dose Vc in treating CRC. Our assays indicate a possible better outcome when certain CRC patients, whose tumors are with high MALAT1 expression, are treated with high-dose intervenes Vc. However, further preclinical tests and clinical trials are still needed.

## Data Availability

The data generated or analyzed during this study are included within the main text and its additional files. Further details in our study are also available from the corresponding author upon reasonable request.

## Abbreviations

Vc: Vitamin C
IVC: Intravenous Vc
CRC: Colorectal cancer
DHA: Dehydroascorbate
GAPDH: Glyceraldehyde 3-phosphate dehydrogenase
LncRNA: Long noncoding RNA
IHC: Immunohistochemistry
qPCR: Quantitative real-time polymerase chain reaction
Ex vivo: Nude mouse xenograft by cancer cell line
MALAT1: Metastasis-associated lung adenocarcinoma transcript 1.

## Supplementary Materials

Supplementary Materials Figure S1: morphology change after treatment with different doses of Vc. A: LS174T was floated after Vc treatment for 4 h in a dose-dependent manner. B: SW480 showed a swelling change after treatment with different doses of Vc for 4 h. Table S1: the primer sequences used in the qPCR assay in this analysis. Table S2: LncRNA profile verified by repeated reports and detected in this article.

## References

[1] Carr A. C., Maggini S. (2017). Vitamin C and immune function. *Nutrients*.

[2] Cameron E., Pauling L. (1976). Supplemental ascorbate in the supportive treatment of cancer: prolongation of survival times in terminal human cancer. *Proceedings of the National Academy of Sciences*.

[3] Creagan E. T., Moertel C. G., O’Fallon J. R. (1979). Failure of high-dose vitamin C (ascorbic acid) therapy to benefit patients with advanced cancer. *New England Journal of Medicine*.

[4] Moertel C. G., Fleming T. R., Creagan E. T., Rubin J., O’Connell M. J., Ames M. M. (1985). High-dose vitamin C versus placebo in the treatment of patients with advanced cancer who have had No prior chemotherapy. *New England Journal of Medicine*.

[5] Padayatty S. J., Sun H., Wang Y. (2004). Vitamin C pharmacokinetics: implications for oral and intravenous use. *Annals of Internal Medicine*.

[6] Stephenson C. M., Levin R. D., Spector T., Lis C. G. (2013). Phase I clinical trial to evaluate the safety, tolerability, and pharmacokinetics of high-dose intravenous ascorbic acid in patients with advanced cancer. *Cancer Chemotherapy and Pharmacology*.

[7] Uetaki M., Tabata S., Nakasuka F., Soga T., Tomita M. (2015). Metabolomic alterations in human cancer cells by vitamin C-induced oxidative stress. *Scientific Reports*.

[8] Mastrangelo D., Massai L., Lo Coco F. (2015). Cytotoxic effects of high concentrations of sodium ascorbate on human myeloid cell lines. *Annals of Hematology*.

[9] Chen Q., Espey M. G., Sun A. Y. (2007). Ascorbate in pharmacologic concentrations selectively generates ascorbate radical and hydrogen peroxide in extracellular fluid in vivo. *Proceedings of the National Academy of Sciences*.

[10] Yun J., Mullarky E., Lu C. (2015). Vitamin C selectively kills KRAS and BRAF mutant colorectal cancer cells by targeting GAPDH. *Science*.

[11] Chen Q., Espey M. G., Sun A. Y. (2008). Pharmacologic doses of ascorbate act as a prooxidant and decrease growth of aggressive tumor xenografts in mice. *Proceedings of the National Academy of Sciences*.

[12] Huebner J., Muenstedt K., Prott F. J. (2014). Online survey of patients with breast cancer on complementary and alternative medicine. *Breast Care*.

[13] American Association for Cancer Research (2014). Parenteral ascorbate is beneficial in ovarian cancer therapy. *Cancer Discovery*.

[14] Vollbracht C., Schneider B., Leendert V., Weiss G., Auerbach L., Beuth J. (2011). Intravenous vitamin C administration improves quality of life in breast cancer patients during chemo-/radiotherapy and aftercare: results of a retrospective, multicentre, epidemiological cohort study in Germany. *In Vivo*.

[15] Siegel R., Desantis C., Jemal A. (2014). Colorectal cancer statistics, 2014. *CA: A Cancer Journal for Clinicians*.

[16] Xie X., Tang B., Xiao Y. F. (2016). Long non-coding RNAs in colorectal cancer. *Oncotarget*.

[17] Yang Y., Zhao L., Lei L. (2017). LncRNAs: the bridge linking RNA and colorectal cancer. *Oncotarget*.

[18] Liu S., Jiang X., Li W., Cao D., Shen K., Yang J. (2016). Inhibition of the long non-coding RNA MALAT1 suppresses tumorigenicity and induces apoptosis in the human ovarian cancer SKOV3 cell line. *Oncology Letters*.

[19] Zou Y., Qin F., Chen J. (2016). sTLR4/MD-2 complex inhibits colorectal cancer in vitro and in vivo by targeting LPS. *Oncotarget*.

[20] Kheirelseid E. A., Chang K., Newell J., Kerin M. J., Miller N. (2010). Identification of endogenous control genes for normalisation of real-time quantitative PCR data in colorectal cancer. *BMC Molecular Biology*.

[21] Santini D., Spoto C., Loupakis F. (2010). High concordance of BRAF status between primary colorectal tumours and related metastatic sites: implications for clinical practice. *Annals of Oncology*.

[22] Van Der Reest J., Gottlieb E. (2016). Anti-cancer effects of vitamin C revisited. *Cell Research*.

[23] Belin S., Kaya F., Duisit G., Giacometti S., Ciccolini J., Fontés M. (2009). Antiproliferative effect of ascorbic acid is associated with the inhibition of genes necessary to cell cycle progression. *PloS ONE*.

[24] Venturelli S., Sinnberg T. W., Niessner H., Busch C. (2015). Molecular mechanisms of pharmacological doses of ascorbate on cancer cells. *Wiener Medizinische Wochenschrift*.

[25] Bordignon B., Chiron J., Fontes M. (2013). Ascorbic acid derivatives as a new class of antiproliferative molecules. *Cancer Letters*.

[26] Espey M. G., Chen P., Chalmers B. (2011). Pharmacologic ascorbate synergizes with gemcitabine in preclinical models of pancreatic cancer. *Free Radical Biology and Medicine*.

[27] Takemura Y., Satoh M., Satoh K., Hamada H., Sekido Y., Kubota S. (2010). High dose of ascorbic acid induces cell death in mesothelioma cells. *Biochemical and Biophysical Research Communications*.

[28] Li H., Ma S. Q., Huang J., Chen X. P., Zhou H. H. (2017). Roles of long noncoding RNAs in colorectal cancer metastasis. *Oncotarget*.

[29] Ji P., Diederichs S., Wang W. (2003). MALAT-1, a novel noncoding RNA, and thymosin *β*4 predict metastasis and survival in early-stage non-small cell lung cancer. *Oncogene*.

[30] Gutschner T., Hämmerle M., Diederichs S. (2013). MALAT1 - a paradigm for long noncoding RNA function in cancer. *Journal of Molecular Medicine*.

[31] Mendell J. T. (2016). Targeting a long noncoding RNA in breast cancer. *New England Journal of Medicine*.

