# Supplementary material for: High-Dose Vitamin C Tends to Kill Colorectal Cancer with High MALAT1 Expression
Source: J Oncol. 2020 Nov 23;2020:2621308. doi: 10.1155/2020/2621308 (PMC7714606; doi:10.1155/2020/2621308)
Supplement: Supplementary Materials — Figure S1: morphology change after treatment with different doses of Vc. A: LS174T was floated after Vc treatment for 4 h in a dose-dependent manner. B: SW480 showed a swelling change after treatment with different doses of Vc for 4 h. Table S1: the primer sequences used in the qPCR assay in this analysis. Table S2: LncRNA profile verified by repeated reports and detected in this article. [file 2621308.f1.docx]

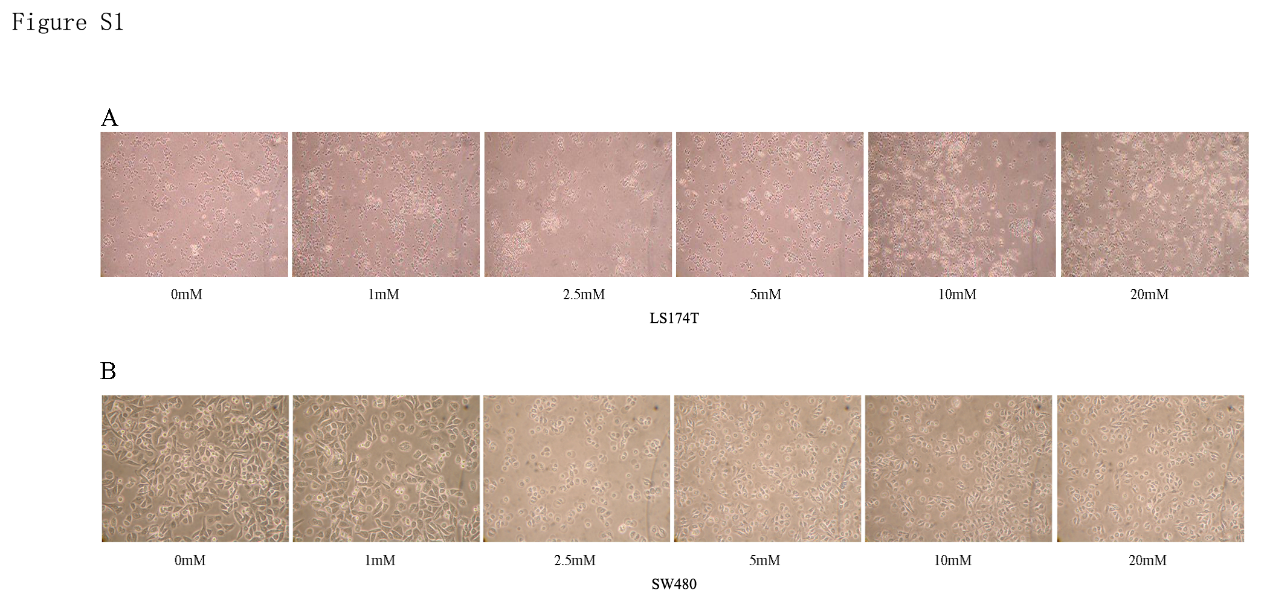
**Figure S1. Morphology change after treated with different dose of Vc**. A. LS174T was floated after Vc treatment for 4h in a dose dependent manner. B. SW480 showed a swelling change after treated with different doses of Vc for 4h.

Table S1. The primer sequences used in the qPCR assay in this analysis

| **Gene Symbol** | **Sense Primer** | **Antisense Primer** |
| --- | --- | --- |
| **PPIA** | 5'- ACCGCCGAGGAAAACCGTGTA-3' | 5'- TGCTGTCTTTGGGACCTTGTCTGC-3' |
| **AFAP1-AS1** | 5'-AATGGTGGTAGGAGGGAGGA-3' | 5'-CACACAGGGGAATGAAGAGG-3' |
| **GAS5** | 5'-AGCTGGAAGTTGAAATGG-3' | 5'-CAAGCCGACTCTCCATACC-3' |
| **CCAT1** | 5'-TCACTGACAACATCGACTTTGAAG-3' | 5'-GGAGAAAACGCTTAGCCATACAG-3' |
| **HOTAIR** | 5'-AAACAGAGTCCGTTCAGTGTCA-3' | 5'-ATTCTTAAATTGGGCCTGGGTC-3' |
| **UCA1** | 5'-CTCTCCATTGGGTTCACCATTC-3' | 5'-GCGGCAGGTCTTAAGAGATGAG-3' |
| **MALAT1** | 5′-GGTAACGATGGTGTCGAGGTC-3′ | 5′-CCAGCATTACAGTTCTTGAACATG-3′ |
| **H19** | 5'-CAACATCAAAGACACCATCGG-3' | 5'-GAGACAGAAGGATGAAAAAGAAGAA-3 |

Table S2 LncRNA profile verified by repeated reports and detected in this article

| **LncRNA** | **Expression pattern in CRC** | **Functions** |
| --- | --- | --- |
| **CCAT1** | Upregulated | CRC development and progression |
| **UCA1** | Upregulated |  |
| **GAS5** | Downregulated |  |
| **CCAT1** | Upregulated | Sustain proliferation |
| **MALAT1** | Upregulated |  |
| **UCA1** | Upregulated |  |
| **GAS5** | Downregulated |  |
| **CCAT1** | Upregulated | Metastasis and invasion |
| **H19** | Upregulated |  |
| **HOTAIR** | Upregulated |  |
| **GAS5** | Downregulated |  |
| **H19** | Upregulated | Clinical application (diagnosis and prognosis) |
| **AFAP1-AS1** | Upregulated |  |
| **GAS5** | Downregulated |  |
